# The Discordance between Network Excitability and Cognitive Performance Following Vigabatrin Treatment during Epileptogenesis

**DOI:** 10.3390/life11111213

**Published:** 2021-11-10

**Authors:** Ming-Chi Lai, Chin-Wei Huang

**Affiliations:** 1 Department of Pediatrics, Chi-Mei Medical Center, Tainan 71004, Taiwan; 860811@mail.chimei.org.tw; 2 Department of Neurology, National Cheng Kung University Hospital, College of Medicine, National Cheng Kung University, Tainan 70101, Taiwan

**Keywords:** vigabatrin, pilocarpine, epileptogenesis, cognition

## Abstract

Vigabatrin (VGB), a potent selective γ-aminobutyric acid transaminase (GABA-T) inhibitor, is an approved non-traditional anti-seizure drug for patients with intractable epilepsy. Nevertheless, its effect on epileptogenesis, and whether this effect is correlated with post-epileptogenic cognitive function remain unclear. Based on lithium-pilocarpine-induced seizure modeling, we evaluated the effect of VGB on epileptogenesis and neuronal damage following status epilepticus in Sprague–Dawley rats. Cognitive evaluations were performed with the aid of inhibitory avoidance testing. We found that VGB could interrupt epileptogenesis by reducing spontaneous recurrent seizures, hippocampal neuronal damage, and chronic mossy fiber sprouting. Nevertheless, VGB did not help with the retention of cognitive performance. Our findings suggest that further research into the role of VGB in epileptogenesis and the treatment of epilepsy in clinical practice is warranted.

## 1. Introduction

Vigabatrin (VGB) is a potent selective γ-aminobutyric acid transaminase (GABA-T) inhibitor with anti-seizure activity which has been approved as a monotherapy for infantile spasms and as an adjunctive therapy for refractory complex partial seizures [1,2,3]. Earlier studies have demonstrated that it increases the levels of the inhibitory neurotransmitter GABA in a dose-dependent manner in the brains of mice and rats and in the cerebrospinal fluid of patients with epilepsy [4,5,6,7]. In addition to its GABAergic effect, we have recently found that VGB modulates neuronal calcium-activated potassium channels, suggesting that it is equipped with various mechanisms of action [8]. In animal models, it has been demonstrated that VGB can abort acute focally evoked pilocarpine-induced seizures [9], pentylenetetrazole (PTZ)-induced seizures [10], and tetrodotoxin-induced infantile spasms [11]. Furthermore, although it has exhibited variable anticonvulsant potential, it generally shows broad-spectrum anticonvulsant activity [4,12].

Despite its beneficial impacts, it is currently unclear whether VGB administered after pilocarpine-induced status epilepticus can interrupt epileptogenesis. It has been found to be detrimental to the recovery process when administered following a focal cortical insult, in contrast to the effects of phenobarbital and diazepam [13]. Another study has reported that VGB results in incomplete protection of the hippocampus, which is widely believed to be involved in temporal lobe seizure activity, and there is evidence that VGB has no influence on the generation of epilepsy [14], suggesting that the expression of the protein glutamic acid decarboxylase is independent of seizures. Finally, a recent study on a TSC/mTOR-dependent epilepsy mouse model has shown that VGB does not prevent epilepsy but significantly delays the onset of seizures and lowers their frequency [15].

Some research suggests the existence of a link between seizure activity and cognitive impairment. One such study, using a kainic acid model of chronic temporal lobe epilepsy (TLE), has highlighted neuropathological features of TLE, including reduced neurogenesis and aberrant migration of newly born neurons and aberrant mossy fiber sprouting in the hippocampus, which appear to be correlated with cognitive impairment [16]. Hippocampal damage is closely related to memory impairment, so the protection of the hippocampus might help to maintain memory processes [14]. Such findings raise the question of whether VGB might impact cognitive function in addition to its role in seizure activity. In fact, it has been noted that increased cognitive risk is associated with persistent or poorly controlled seizures [16]. Nonetheless, the cognitive effects of VGB have been incompletely evaluated and remain inconclusive [17,18]. Furthermore, the clinical effects of VGB, which are relevant to mitigating the risk of both cognitive impairment and seizure activity, have proven to be either unclear or favorable depending on the etiology and preexisting developmental profile of seizure activity [19,20].

The current evidence suggests that more research is required to ascertain the effectiveness of VGB. For example, the relationships between cognitive performance, seizure severity, and the effects of VGB on animals with chronic pilocarpine-induced epilepsy remain to be determined. The possibility that spontaneous recurrent seizures and network excitability following pilocarpine-induced epileptogenesis have an impact on cognitive function must also be verified. As a result, in this study, we attempt to determine if the administration of VGB following pilocarpine-induced status epilepticus can help prevent epileptogenesis and preserve cognitive performance.

## 2. Materials and Methods

All experiments were conducted in accordance with the specifications proposed by the Experimental Ethics Committee of National Cheng Kung University (NCKU). The procedures for animal experimentation were reviewed and approved by the Institutional Animal Care and Use Committee, NCKU (Approval No.: 109218).

### 2.1. Animals

Adult male Sprague–Dawley rats weighing 180–200 gm were purchased from NCKU. They were housed in the university’s Animal Center and allowed free access to water and a pelleted rodent diet (Richmond Standard; PMI Feeds, St. Louis, MO, USA). A cycle of alternating 12-h periods of light and dark was used to ensure the animals’ circadian rhythms functioned under normal, environmentally relevant conditions [21]. Efforts were made to keep the number of rats used to a minimum.

### 2.2. In Vivo Experiments

Grouping

Lithium-Pilocarpine Seizure Modeling

On day 1, the rats were injected with lithium chloride (3 meq/kg; i.p.), and methylscopolamine (25 mg/kg; s.c.) prior to their experiencing pilocarpine-induced seizures (60 mg/kg; s.c.). The behavioral characteristics of the rats during the epileptic seizures were similar to those reported earlier in our laboratory and elsewhere [22,23,24], and the behavioral stages of the seizures were evaluated based on the Racine scale [25]. Over the following 15 to 20 min, they exhibited mouth and facial movements, head bobbing and nodding, scratching, masticatory automatisms, and exploratory behavior (stages 1–2). Episodes of myoclonic movements of the head and bilateral forelimbs (stage 3) started at 20 to 25 min and progressed to status epilepticus (stages 4–5), with rearing and falling at about 50 min after the pilocarpine injection. To measure seizure latency, the time intervals from the administration of pilocarpine to the onset of overt seizure behaviors (stage 3) were calculated. Diazepam (5 mg/kg, i.p.) was administered to diminish the intensity of the seizures if status epilepticus lasted for 90 min. Status epilepticus-related mortality was recorded during the first 24 h after the initial onset. All the rats were monitored for the first 24 h after status epilepticus, and supportive care was implemented, including maintaining body temperature, feeding, and providing adequate hydration.

**Figure 1:**
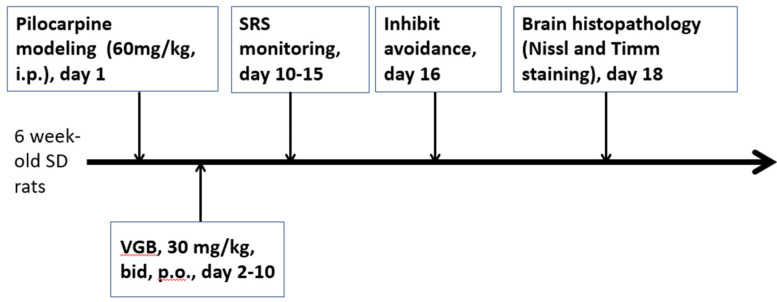
The experimental protocol. SD: Sprague–Dawley; VGB: vigabatrin; SRS: spontaneous recurrent seizure.

The rats were then divided into an experimental group (VGB) (orally fed 30 mg/kg of VGB twice daily for 7 consecutive days, days 2–9) and a control normal saline (NS) group (orally fed normal saline daily for 7 consecutive days). Each group contained 7 animals in each set of experiments. After this latent phase of epileptogenesis (days 2–9), the rats gradually developed chronic epilepsy characterized by spontaneous recurrent seizures (SRS). We started monitoring the seizure frequency in the rats on days 10–15 after status epilepticus. Each rat was monitored with a video camera set above the cage 8 h per day for 5 consecutive days [24,26]. All monitoring of the SRS was conducted by a trained technician blinded to the experimental design. The videos were watched to identify any evidence of seizure behavior (running, jumping, rearing, lordosis, an erect tail, and so on). If any seizure-like activity was seen, the video was stopped and viewed again to evaluate the behavior for possible seizures. The experimental protocol is shown in Figure 1.

### 2.3. Inhibitory Avoidance Task

A single-trial inhibitory avoidance (IA) task, which is a hippocampus-dependent learning task, was used to evaluate the different stages of memory in rats on day 16, after the monitoring of SRS. The apparatus consisted of one illuminated compartment and one dark compartment. A shock generator was connected to the floor of the dark compartment. Before the experiment, the rat was kept in a dimly lit room for 1 h to adjust to the brightness level and environment. During the training phase, the rat was placed in the illuminated compartment facing away from the door. As the rat turned around, the door was opened. When the rat entered the dark compartment, the door was closed, and the rat was given a 1.0 mA/1-s shock. The reaction to the shock was identified as flinch, vocalization, or locomotion. The rat was then removed from the alley and returned to its home cage. A retention test was conducted 1, 3, and 24 h after the training phase to evaluate short-term, intermediate, and long-term memory, respectively. This test involved again placing the rat in the illuminated compartment, after which its hesitancy to step into the dark compartment was recorded as a measure of retention. Rats that did not enter the dark compartment within 600 s were removed from the alley.

### 2.4. Histopathology

#### 2.4.1. Cresyl Violet Staining

On day 18, the rats were anesthetized with an overdose of pentothal (60 mg/kg; i.p.), and then their brains were removed and stored at −80 °C. The next step involved fixing coronal sections (20-μm thick) of the hippocampus in formaldehyde in order to be stained with cresyl violet, as has been described in previous research [24,27]. The hippocampal subfields were defined by means of an imaginary line connecting the blade tips of the granule cell layer, which made it possible to isolate the cornu ammonis (CA) and separate its regions: CA3c (medially) from CA3b (laterally), and CA2 from CA1 [23,24]. The cresyl violet-stained sections then underwent a gross examination for indications of damage in the hippocampus. In order to assess neuron damage, the cells were counted using Nissl-stained sections (10-μm thick), and the images were magnified (×400) using a computerized image analysis system (Image Plus 2.0; Motic, Richmond, British Columbia, Canada) in order to facilitate the counting. The severity of neuron damage in different subfields of the hippocampus was scored semi-quantitatively as follows: 0 = no damage, 1 = less than 10%, 2 = between 11% and 50% neuron loss, and 3 = equal to or greater than 50% neuron loss [28,29]. Scores for the VGB and NS groups were obtained by an investigator blinded to the study design, and then an average score was calculated for each group.

#### 2.4.2. Timm’s Staining

On day 18, after the rats’ brains had been removed, coronal sections (20-μm thick) were cut through the entire hippocampus on a freezing microtome. Timm staining was performed on every sixth section [30] from the septal region to the temporal region of the hippocampus (the region between 2.8 and 3.8 mm posterior to the bregma). The sections were processed in the dark for 10–45 min in 200 mL of a solution containing 5.1 g of citric acid, 4.7 g of sodium citrate, 3.47 g of hydroquinone, 212.25 mg of AgNO_3_, and 120 mL of 50% arabic gum. We used a semi-quantitative scale to evaluate the degree of mossy fiber sprouting in the pyramidal cell layer of the CA3 hippocampal region, in the granular cell layer and inner molecular layer of the dentate gyrus, and in the infra-pyramidal mossy fiber area between CA3 and the dentate gyrus [23,24,27]. The scale scores included: 0 = no granules, 1 = occasional discrete granule bundles, 2 = occasional-to-moderate granules, 3 = prominent granules, 4 = prominent near-continuous granule bands, and 5 = continuous or nearly continuous dense granule bands.

### 2.5. Drugs and Solutions

VGB, scopolamine, and pilocarpine were purchased from Sigma–Aldrich (St. Louis, MO, USA). All other chemicals, unless otherwise noted, were locally purchased and of reagent grade.

### 2.6. Statistical Analysis

In this paper, the results of the analyses of the continuous data are expressed as means ± standard error of the mean (SEM), unless otherwise indicated. With the statistical significance set at *p* < 0.05, the continuous variables were assessed using either *t*-tests or a one-way analysis of variance (ANOVA) (SPSS 17.0; SPSS Institute, Chicago, IL, USA), followed by Fisher’s least significant difference (LSD) test. The Shapiro–Wilk test of the normality of the distribution of the dataset was performed, and when data were not normally distributed, analyses were done using the ANOVA (the Kruskal–Wallis H test), followed by Dunn’s multiple comparison test. Analyses were also done using χ^2^ tests, the Yates χ^2^ test, and Fisher’s exact probability test with the nominal variables.

## 3. Results

### 3.1. VGB-Treated Rats Had Fewer Spontaneous Recurrent Seizures

**Figure 2:**
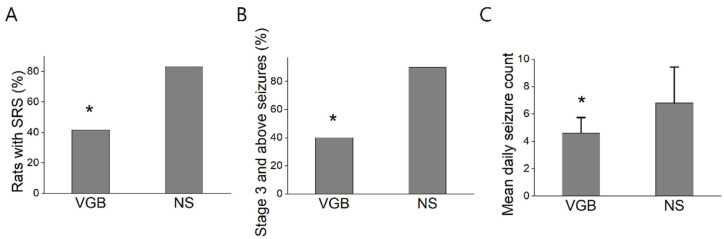
VGB-treated rats had fewer spontaneous recurrent seizures. (**A**) The VGB-treated group had fewer rats with SRS, compared to the normal saline group (VGB: 41.67%; NS: 183.33%, * *p* < 0.05). (**B**) The VGB group had fewer severe seizures (stage 3 and above), as compared to the NS group (VGB: 40%; NS: 90%, * *p* < 0.05). (**C**) The mean count of daily seizures for the VGB group was significantly lower than that for the NS group (VGB: 4.6 ± 1.14; NS: 6.8 ± 2.62, * *p* < 0.05) (*N* = 7 in each group).

After a latent phase of one week following status epilepticus, the rats developed spontaneous seizures. These SRS were characterized by spontaneous convulsions [31] with head nodding, forelimb clonus, rearing, and falling. The mean number of daily spontaneous seizures exhibited per rat was calculated based on regular daily 4-h visual monitoring from 14 to 28 days after pilocarpine-induced status epilepticus. The rats were evaluated in their respective group, with the VGB-treated group having fewer rats with SRS, compared to the normal saline group (VGB: 5/12, or 41.67%; NS: 10/12, or 83.33%, *p* = 0.03) (see Figure 2A). The rats in the VGB group had fewer severe seizures (stage 3 and above), compared to the NS group (VGB: 2/5, or 40%; NS: 9/10, or 90%, *p* = 0.03) (see Figure 2B). The mean number of daily seizures in the VGB group was significantly smaller than in the NS group (VGB: 4.6 ± 1.14; NS: 6.8 ± 2.62, *p* = 0.04) (See Figure 2C). In sum, fewer rats in the VGB-treated group exhibited SRS, and this group showed fewer severe seizures and daily seizures.

### 3.2. The VGB-Treated Rats Had Less Post-Status Epilepticus Chronic Hippocampal Damage

**Figure 3:**
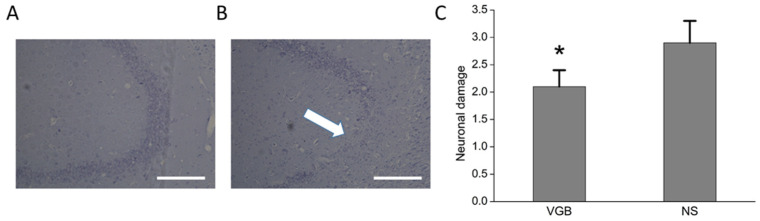
VGB-treated rats had less post-status epilepticus chronic hippocampal damage. The VGB group (**A**) had significantly less neuron loss in the hippocampal CA3 region than was the case in the NS group (**B**) (the arrow shows the cresyl violet staining). A blind semi-quantitative analysis (**C**) showed that the VGB group had significantly less hippocampal neuronal damage compared with the NS group (* *p* < 0.01) (*N* = 7 in each group). The scale bar = 200 μM.

After epileptogenesis, the cresyl violet staining showed that the VGB group had significantly less neuron loss in the hippocampal CA3 field (see Figure 3A) than the NS group (see Figure 3B). A blind semi-quantitative analysis showed that the VGB group had significantly less hippocampal neuronal damage compared with the NS group (VGB: 2.1 ± 0.3; NS: 2.9 ± 0.4, *p* < 0.01) (See Figure 3C). In conclusion, the VGB-treated group exhibited less chronic hippocampal damage post-pilocarpine-induced status epilepticus.

### 3.3. VGB-Treated Rats Had Less Aberrant Mossy Fiber Sprouting

**Figure 4:**
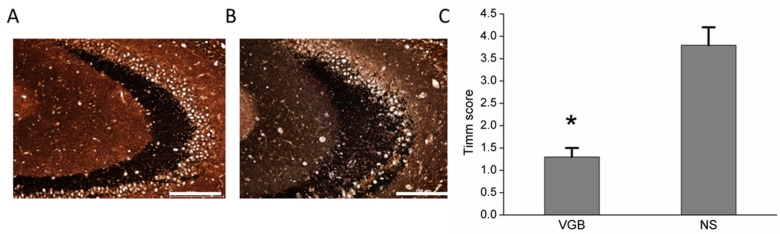
VGB-treated rats had less mossy fiber sprouting. Timm’s staining (**A**,**B**) showed significantly fewer mossy fibers sprouting in the hippocampal CA3 region of the VGB group (**A**) than in the NS group (**B**). The Timm’s score (**C**) for VGB vs. NS were significant, * *p* < 0.05) (*N* = 7 in each group). The scale bar = 200 μM.

Eighteen days following status epilepticus, Timm staining (see Panels A and B in Figure 4) showed significantly fewer dense mossy fibers sprouting in the hippocampal CA3 region of the VGB rats than in the NS rats (Timm scores of VGB: 1.3 ± 0.2, and NS: 3.8 ± 0.4, *p* < 0.05) (see Panel C in Figure 4). Therefore, the VGB-treated rats showed less sprouting of dense mossy fibers in the hippocampus.

### 3.4. VGB-Treated Rats Did Not Preserve Inhibitory Avoidance Test Performance

**Figure 5:**
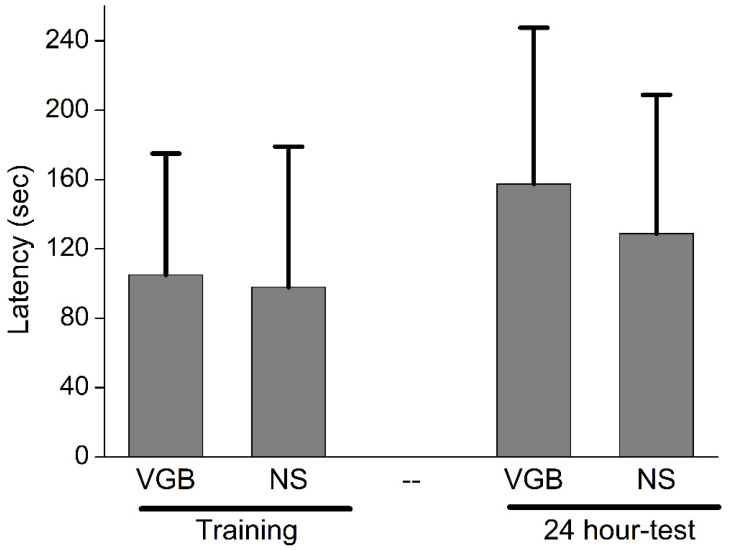
VGB-treated rats did not preserve inhibitory avoidance test performance. The duration of the hesitancy to enter the dark compartment during training and after training did not differ significantly between the VGB and NS groups (VGB vs. control, *p* = 0.18).

The duration of the initial hesitancy to enter the dark compartment during training did not significantly differ between the two groups. After training, the time elapsed before the rats entered the dark compartment also did not differ significantly between the VGB and NS groups, although the former tended to exhibit relatively more retention time of the avoidance response after training (VGB: 157.5 ± 90 s; NS: 128.9 ± 80 s, *p* = 0.18) during the chronic stage after pilocarpine-induced status epilepticus (See Figure 5). In conclusion, the cognitive performance of the rats in the VGB-treated group was not preserved during the inhibitory avoidance test.

## 4. Discussion

In this study, we demonstrated that VGB was effective in ameliorating lithium-pilocarpine-induced epileptogenesis, and in reducing hippocampal neuronal damage and mossy fiber sprouting. Nevertheless, VGB did not allow for retention of cognitive performance following epileptogenesis in our inhibitory avoidance model.

It has been reported that VGB administered to patients with epilepsy showed few adverse effects on cognition [32,33] and fewer adverse effects than carbamazepine on cognitive processes, including memory, psychomotor speed, and flexibility in mental processing [34]. It is generally regarded as harmless to the cognitive performance when used as an antiepileptic drug to treat patients with newly diagnosed and intractable epilepsy. In our study, however, it did not help to retain cognitive functions during the epileptogenesis stage, although it did attenuate spontaneous recurrent seizures and reduce acute neuronal damage and aberrant excitatory networks. This suggests that the frequency of recurrent seizures, a common indicator of cognitive function, is probably not actively involved in determining the cognitive activity associated with using VGB to treat seizures.

Another possible explanation is that significant neuronal damage occurs immediately after status epilepticus, leading to severe cognitive deficits. Although subsequent treatment with VGB attenuated neuronal hyperexcitability, leading to less frequent seizures and secondary neuronal damage, the altered functioning could not be restored, at least during the epileptogenesis stage.

GABAergic interneurons play a critical role in higher brain functions, and signaling from interneurons to astrocytes normally sustains important cortical information processing and complex behaviors [35]. In an epileptic network with extensive aberrant neuronal sprouting and altered ionic mechanisms, the effect of VGB on GABAergic activity probably makes it impossible for GABAergic interneurons to exert normal signaling and behavioral responses. Furthermore, the stage of epileptogenesis is different from the stage of chronic static epilepsy, and it is probable that the wide spectrum of cognitive deficits observed in chronic epilepsy cannot be attributed to seizures and antiepileptic drugs alone. Etiology and aberrant synaptic transmissions may also play important roles in cognitive impairment [18,36].

One study found no anti-epileptogenic effects of VGB in an animal model examining kainic acid-induced seizures [37], although the correlation between the severity of neuronal damage and the extent of mossy fiber sprouting was similar to that found in our study. Probable reasons for their findings are the severe hippocampal damage and inter-animal variability seen in their model. Earlier findings have shown that VGB delayed the development of kindling [38], which is in line with our findings. Another study has shown VGB to have protective effects in the CA3 pyramidal cell layer in a pilocarpine model [14] although the latency to seizure was not altered.

The possibility has been raised that mossy fiber sprouting disrupts the functioning of hippocampal neuronal circuits and contributes to cognitive impairment in models of temporal lobe epilepsy [39]. Earlier studies evaluating hippocampal neurogenesis have not found consistent evidence of its impact on cognitive performance, and those examining mossy fiber sprouting in temporal lobe epilepsy suggest that the link between mossy fiber sprouting and cognitive function remains inconclusive. Interestingly, the use of valproic acid to block seizure-induced neurogenesis has been shown to have a protective effect against cognitive impairment [40]. Our finding that VGB did not help retain cognitive performance although it attenuated mossy fiber sprouting and epileptogenesis in our pilocarpine model is supported by a recent study in which an overall reduction in the number of abnormal neuronal integrations, including mossy fiber sprouting, in epileptic rats did not produce any difference in their performance on the Morris water maze test compared with that of rats whose neurogenesis had not been ablated [41].

It has been reported that, following pilocarpine-induced status epilepticus, only epilepsy-prone rats showed accelerated forgetting rates and reduced learning rates during the Morris water maze task, compared to both rats that did not develop epilepsy and rats used as controls, suggesting that cognitive deficits functioned as a biomarker of epileptogenesis in this rat model of epilepsy [42]. However, our study using an inhibitory avoidance task does not support this finding. Both our VGB group and our control group showed similar cognitive profiles, with differences in epileptogenicity. Whether this result is related to the severity of the neuron loss in both groups, as we mentioned earlier, or to the ratio of normal versus abnormal/epileptic neurons remains to be determined. A recent study has shown that VGB, with the aid of inhibitory avoidance and open-field tasks, did not affect short-term memory or long-term memory, but it impaired exploration and locomotion performance [43], probably as a result of its sedative effect. Whether this effect contributed to the lack of difference in inhibitory avoidance behavior in our study remains to be determined in future studies.

According to the literature, some patients on VGB therapy develop visual field defects. It has been suggested that around one third of them experience such loss of vision [44]. Nonetheless, visual acuity and color vision seem to remain stable in all these patients regardless of changes in their field of vision [45], and it has been suggested that VGB-associated visual field defects are an idiosyncratic drug reaction within the neurosensory retina [44,45]. In the context of our study, if visual field defects had affected cognitive performance, we would have seen differences between the VGB-treated group and the NS group during the training phase of our inhibitory avoidance test. However, their performance at this point was similar, and their memory retention measured later did not show any significant differences between the groups. Therefore, we believe that the possible presence of visual field deficits in the rats would not have affected their cognitive performance during this task.

There are multiple factors affecting cognitive performance in patients with epilepsy [46], and there is contradictory evidence of a relationship between seizure control and/or structural damage, on the one hand, and cognitive performance, on the other [47]. Although clinical epileptic seizures tend to resolve on their own or markedly improve, cognitive outcomes are not always favorable, and a structural etiology typically confers a poor prognosis [48]. In addition, some changes in the brain regions that are important for behavior and cognition, as well as altered thalamofrontal neurodevelopment occurring during brain maturation have been shown to be independent of seizure variables [48,49]. Finally, our study findings support the notion that the severity of neuronal damage and the reduced frequency of recurrent seizures do not necessarily correlate with better cognitive performance during the epileptogenesis stage.

## 5. Conclusions

Our study with rats showed VGB to be effective in interrupting epileptogenesis and in reducing hippocampal neuronal damage and mossy fiber sprouting, but not in retaining cognitive performance. It should also be noted that, although our data are not sufficient to guarantee similar results if VGB is administered to humans with different types of epilepsy, clinical attention to the use of VGB therapy for patients with epilepsy is recommended.

## Data Availability

The data are available upon request made to the correspondence author.
